# Liver Transplant for Acute Cholestatic Crisis in Sickle Cell Disease

**DOI:** 10.3389/ti.2025.14086

**Published:** 2025-03-27

**Authors:** Sabrina Welland, Jan Christopher Kamp, Björn Hartleben, Richard Taubert, René Abu Isneineh

**Affiliations:** ^1^ Department of Gastroenterology, Hepatology, Infectious Diseases and Endocrinology, Hannover Medical School, Hannover, Germany; ^2^ Department of Respiratory Medicine and Infectious Diseases, Hannover Medical School, Hannover, Germany; ^3^ Institute of Pathology, Hannover Medical School, Hannover, LowerSaxony, Germany

**Keywords:** liver transplant, acute liver failure, exchange transfusion, sickle cell disease, sickle cell-associated intrahepatic cholestasis

Dear Editors,

We here report on the management of a 54-year-old female patient who developed severe liver and multi-organ failure due to a severe veno-occlusive crisis in the context of sickle cell disease (SCD) and eventually underwent high urgency transplantation.

After presentation to the emergency department with clinical signs of severe acute liver failure (ALF), a heterozygous sickle cell disease with recurrent symptomatic hemolytic crisis without any preexisting liver disease was diagnosed. The laboratory markers of liver function on admission are outlined in Table 1. In the past, a splenectomy and femoral head replacement after aseptic bone necrosis had been performed due to severe hemolytic crisis.

**TABLE 1:** Laboratory results on the day of admission (d0), on the day of high urgency listing (d2), immediately before transplantation (d3, after plasmapheresis), 24 h after transplantation (d4), and 6 days after transplantation (d10).

	Admission (d0)	Transplant listing (d2)	Pre-transplant (d3)	Post-transplant (d4/d1 post Tx)	Post-transplant (d10/d6 post Tx)
Creatinine (45–84 μmol/L)	97	58	48	75	49
ALT (<31 U/L)	1,445	1,100	603	3,254	36
AST (<34 U/L)	1,111	999	611	2,448	318
GLDH (<5 U/L)	83	51	35	5,199	17
Bilirubin (2–21 μmol/L)	136	220	177	58	47
AP (35–104 U/L)	165	89	95	96	201
INR (0.9–1.25)	4.21	4.26	1.34	1.35	1.12
Ammonia (11–51 μmol/L)	150	92	56	Nm	Nm
FV (70%–180%)	11.9	10.2	59.1	40.8	Nm
MELD	31	32	19	-	-
Lactate (<2.4 mmol/L)	8.4	1.6	2.0	0.7	0.8
LDH (<247 U/L)	874	539	341	Nm	nm
HbS (%)	83	10.7	Nm	Nm	13.2

ALT, Alanine aminotransferase; AST, Aspartat aminotransferase; AP, Alkaline phosphatase; FV, Factor V; LDH, Lactate hydrogenase; GLDH, Glutamate dehydrogenase; MELD, Model of Endstage Liver Disease; nm, not measured; HbS, Sickle cell hemoglobin.

A liver biopsy showed sinusoidal obstruction and congestion due to sickle cell aggregates (Figure 1) with no evidence of drug-toxic damage or advanced fibrosis. Sickle cell hepatopathy in sickle cell-associated intrahepatic cholestasis (SCIC) with ALF was diagnosed based on the histological picture, the clinical presentation, and the high sickle cell hemoglobin (HbS content (83%).

**FIGURE 1:**
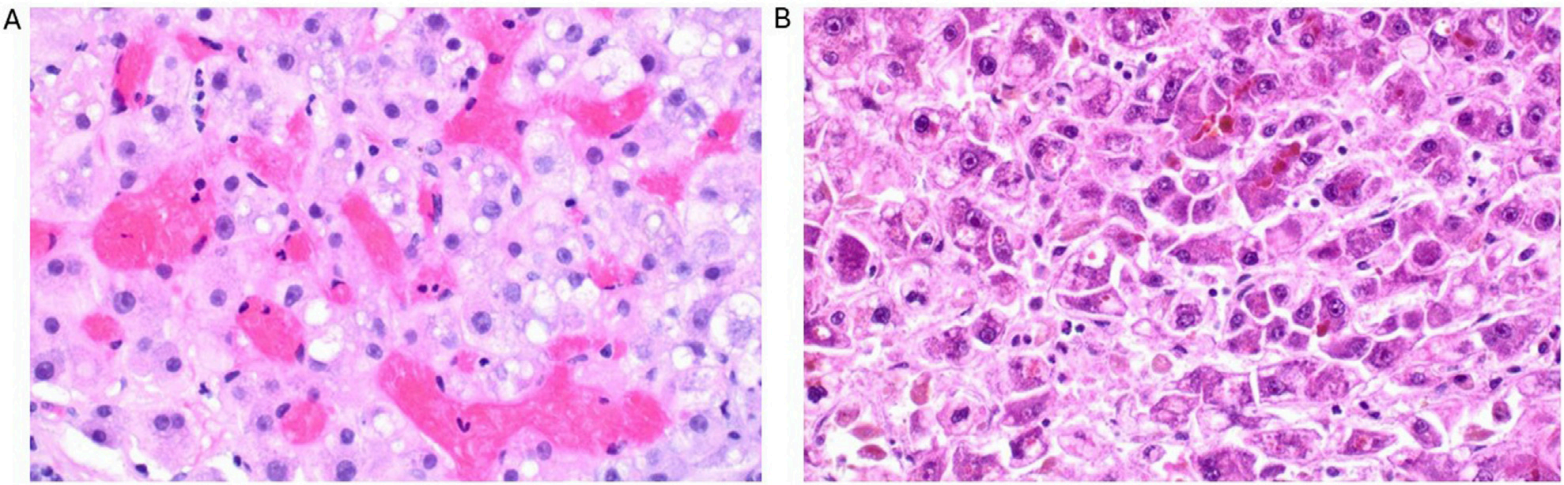
Histological findings of the liver biopsy (1 day after admission) with sinusoidal obstruction and congestion due to sickle cell aggregates with hepatocyte swelling and numerous single cell deaths **(A)** as well as the liver explant (3 days after admission) with significantly more pronounced necrosis compared to the prior biopsy **(B)**.

Supportive drug therapy was initiated for ALF by means of continuous intravenous administration of ornithine aspartate, lactulose enemas, and continuous acetylcysteine infusion. In addition, the sickle cell crisis was treated with continuous glucose infusion and exchange transfusions, which lowered the HbS content to <20%. Due to the pre-existing immunization as result of the extensive pre-transfusions with detection of irregular erythrocyte antibodies, erythrocyte concentrates without anti Cw and anti E were administered.

Despite these measures, the patient developed progressive liver failure (Table 1), which ultimately met the requirements for “high urgency” listing for liver transplantation, which was arranged after excluding contraindications 2 days after admission. In the course, the patient developed progressive multi-organ failure with severe encephalopathy, coagulopathy, lactic acidosis, and circulatory insufficiency. In terms of a bridge-to-transplant strategy, therapeutic plasma exchange and human albumin dialysis were initiated to treat the ALF and the hepatic encephalopathy, respectively, resulting in sufficient stabilization. A suitable donor organ was available 3 days after admission. The transplantation of the whole organ was performed in one stage without complications. The arterial anastomosis was performed as a branch patch of the recipient and donor gastroduodenal artery due to the existing anatomical conditions. Anastomization of the bile duct was performed as an end-to-end anastomosis. The cold ischemia time was 498 min.

In the postoperative course, the HbS percentage rose to 41% necessitating further exchange transfusions. The patient suffered from prolonged postoperative delirium with inconspicuous neuro imaging results. In order to prevent further sickle cell crises and organ complications, a concept of a close HbS surveillance was initiated in close cooperation with the local treating hematologist to assure a sufficient lowering of HbS percentages below 20%, for which a kimal catheter was inserted prior to discharge. Prophylactic anticoagulation was administered during the inpatient stay, which was discontinued on discharge.

No complications, particularly no further sickle cell crises, occurred over the further course under close hematological care with regular exchange transfusions, hydroxycarbamide treatment, and specialized follow-up at our liver transplant outpatient department.

At the last presentation, 18 months after liver transplantation, the graft function was unrestricted with no evidence of advanced hepatic fibrosis (acoustic radiation force impulse imaging elastography 0.96 m/s). The patient was on combined immunosuppression with tacrolimus (trough level aim 5–8 ng/mL) and mycophenolate (500 mg b.i.d.), had no history of acute rejection episodes, and will now be included in our surveillance biopsy guided personalized immunosuppression program.

Vaso-occlusive crises with secondary organ failure including hepatobiliary damage can occur in the context of SCD [1]. SCIC is a rare and severe form of sickle cell hepatopathy leading to local hypoxia with infarction and ballooning of the hepatocytes via sickle cell aggregates in the liver sinusoids. This can cause severe cholestasis and, in very few instances, result in acute liver failure (ALF) [2]. The number of liver transplantations due to acute hepatic sickle cell crisis with irreversible organ failure is very low, so there is only limited experience.

Levesque et al. summarized 21 published cases of liver transplantations in SCD patients and formulated recommendations for the pre- and postoperative management [1]. The 5-year overall survival rate after transplantation was 65% in this cohort, comparable to that of recipients who undergo liver transplantation due to other diseases, while the rate of significant cerebral, micro- and macroangiopathic as well as septic complications was higher. The authors emphasized the need for close pre- and postoperative monitoring of hemoglobin S (HbS) levels for the early detection of imminent vascular occlusions. In addition, given the increased incidence of cerebral bleeding events and vascular occlusions in this patient population, it was recommended to clarify occuring neurological symptoms via neuro imaging with low threshold. Additionally, prophylactic anti-infective therapy was suggested in the light of increased sepsis rates.

The patient cohort reported was very heterogeneous: only 7 patients underwent liver transplantation in the context of SCIC-associated liver failure, while the majority of patients was electively listed for transplantation and/or required transplantation due to another end-stage liver disease, so that the transplantation did not take place in an acute sickle cell crisis.

In the few cases described in the literature, vascular complications such as thrombosis and bleeding occurred significantly more frequently after liver transplantation in SCIC [1, 2]. The patients with ALF and SCIC described by Levesque et al. predominantly had a homozygous mutation in the HbS gene and suffered from early and fatal vascular complications after transplantation despite HbS <20%. Patients with SCIC without early vascular complications, like the patient described in our case, had a HbS-β°-thalassaemia with consistent control of the HbS level to <20% using hydroxycarbamide and exchange transfusions. We support the statement of Levesque et al. that this secondary prophylaxis of new sickle cell crises plays a key role in the post-transplant course in order to avoid vascular complications.

Another important factor for beneficial post-transplant courses is the regular re-evaluation of immunosuppression, particularly due to pre-existing alloimmunization from multiple transfusions prior to transplantation, which can contribute to rejections. A low-threshold transplant biopsy in the event of elevated transaminases can help to detect and treat rejections at an early stage.

Taken together, after transplantation of acute liver failure in SCIC, low-threshold and consistent management of postoperative sepsis episodes and neurological complications is crucial. Exchange transfusions are essential if the HbS level is >20% to avoid vascular complications, as is a low-threshold transplant biopsy in the event of an increase in transaminases.

## Data Availability

The original contributions presented in the study are included in the article/supplementary material, further inquiries can be directed to the corresponding author.
